# Supplementary material for: Caregivers’ View of Socio-Medical Care in the Terminal Phase of Amyotrophic Lateral Sclerosis—How Can We Improve Holistic Care in ALS?
Source: J Clin Med. 2022 Jan 4;11(1):254. doi: 10.3390/jcm11010254 (PMC8745628; doi:10.3390/jcm11010254)
Supplement: Supplementary file 1 [file jcm-11-00254-s001.zip › jcm-1520073-supplementary.pdf]

Table S1. Duration and characteristics of specialized palliative care in our sample.

Number of Patients	Description of SPC	Details
n = 29	None	-
n = 7	SOPC team, home visits; prescribed at the Motoneuron disease specialist center	prescription: mean 120 (3–420) * days prior death; in contact: mean 30 (3–75) * days prior death
n = 7	SOPC team, home visits; prescribed by other actors	in contact: mean 45 (14–240) * days prior death
n = 3	SOPC team, home visits	no further details available
n = 1	SOPC team, nursing home visits	first contact 14 months prior death, regularly since 4 months prior death
n = 1	Hospice last 6 months	plus sporadic SOPC contact before
n = 1	Hospice last 5 days	-
n = 1	Hospice last 4 days	plus sporadic SOPC contact during last two years before

* Median (range); SOPC; specialized outpatient palliative care.

Table S2. Characteristics of disease and wellbeing in different care settings; *n* = 49.

	**Family Care (*n* = 16)**	**Family Care and Nursing Service (*n* = 20)**	**Inpatient Care Setting (*n* = 13)**	***p***
BSFC-S (*n* = 46) [a]	9.5 ± 6.6	10.2 ± 6.3	15.1 ± 7.2	0.069 *
ALSFRS-R prior death [a]	18.3 ± 6.4	12.0 ± 8.7	11.3 ± 8.4	**0.032 ***
Patients‘ age (years) [a]	68.6 ± 9.4	70.4 ± 7.9	74.9 ± 7.5	0.129 [x]
Duration MND diagnosis to death (months) [a]	26.8 ± 37.6	30.1 ± 28.1	26.9 ± 29.6	0.936 [x]
Gender caregivers m:f [b]	7:9 (44:56)	7:13 (35:65)	5:8 (38:62)	0.866 [o]
SPC yes:no [b]	7:9 (44:56)	11:9 (55:45)	4:9 (31:69)	0.390 [o]
Time spent for care (hours per week) [a]	78.1 ± 47.8	50.7 ± 42.7	0.9 ± 3.0	<0.0001 [x]
Time spent together, excluding care (hours per week) [a]	28.6 ± 21.4	25.9 ± 28.0	8.3 ± 7.0	**0.012** [x]

[a] Data are presented as mean ± standard deviations; [b] data are presented as n (%); * ANOVA; [x] Kruskal–Wallis test; [o] chi^2; ALSFRS-R, Amyotrophic Lateral Sclerosis Functional Rating Scale-Revised; BSFC-s, Burden Scale for Family Caregivers short version; SPC, specialized palliative care; The bold means p-values reached significance.
